# Very low calorie diets are associated with transient ventricular impairment before reversal of diastolic dysfunction in obesity

**DOI:** 10.1038/s41366-018-0263-2

**Published:** 2018-11-21

**Authors:** Jennifer J. Rayner, Ines Abdesselam, Mark A. Peterzan, Ioannis Akoumianakis, Nadia Akawi, Charalambos Antoniades, Jeremy W. Tomlinson, Stefan Neubauer, Oliver J. Rider

**Affiliations:** 1 Oxford Centre for Clinical Magnetic Resonance Research, Division of Cardiovascular Medicine, Radcliffe Department of Medicine, University of Oxford, Oxford, UK; 2 Division of Cardiovascular Medicine, Radcliffe Department of Medicine, University of Oxford, Oxford, UK; 3 Oxford Centre for Diabetes, Endocrinology and Metabolism (OCDEM), NIHR Oxford Biomedical Research Centre, University of Oxford, Oxford, UK

**Keywords:** Cardiovascular diseases, Nutrition therapy, Obesity

## Abstract

**Objectives:**

Very low calorie diets (VLCDs) are effective at clearing hepatic steatosis and improving insulin sensitivity. Whilst long-term weight loss is beneficial to the cardiovascular system, the acute elevation in fatty acids during caloric restriction is potentially detrimental to cardiac metabolism and function. We sought to investigate any cardiovascular changes occurring over the course of a modern VLCD regime, alongside the expected peripheral metabolic improvements.

**Methods:**

25 obese volunteers (BMI 36.8 ± 5.8 kg/m^2^) underwent magnetic resonance imaging, echocardiography, metabolic profiling, and bio-impedance analysis before 1 and 8 weeks following a VLCD (800 kcal/day). Results were compared to 15 age- and sex-matched controls.

**Results:**

After 1 week of VLCD, despite only modest weight loss, significant drops occurred in liver fat and insulin resistance (HOMA-IR; by 14–50%, all *p* < 0.01). In contrast, myocardial triglyceride content (MTGC) increased (by 48%, *p* = 0.030), and was associated with deterioration in both systolic (LVEF by 4%, *p* = 0.041) and diastolic function (*e*/*e*′ 8.6 ± 1.4 to 9.4 ± 1.7, *p* = 0.019). Aortic stiffness also increased by 35% (*p* = 0.015).

At 8 weeks, liver steatosis and visceral fat were lower than baseline (by 20–55%, *p* < 0.001), and peripheral metabolic improvements continued. MTGC also fell to below baseline (1.5 ± 0.6 vs 2.1 ± 1%, *p* = 0.05) with improved myocardial function (*e*/*e*′ 8.6 ± 1.4 to 7.5 ± 1.5, *p* = 0.003).

**Conclusions:**

Whilst VLCDs result in dramatic improvements in insulin resistance, they are associated with transient but significant cardiovascular functional decline, which may have an impact on those with the coexisting cardiac disease. However, after 8 weeks, the diet was associated with normalisation of cardiac function, suggesting they may form a potential therapeutic intervention for diastolic dysfunction in obesity and diabetes.

## Introduction

In the 1970s, severely calorie-restricted diets became a commonplace means of losing weight. However, in 1978 a Food and Drug Administration investigation of 800,000 patients adhering to liquid protein very low calorie diets (VLCDs) reported 46 deaths [1]. Following this, sales of liquid replacement meals plummeted and ‘crash’ dieting was widely regarded as dangerous.

With increasing rates of obesity and associated type II diabetes, VLCDs have again become popular. However, modern VLCD plans provide nutritionally balanced meal replacement, and have shifted focus towards effectively reversing type II diabetes [2]. Profound caloric restriction leads to rapid reductions in liver fat, driven by hepatic lipolysis and reduced ectopic triglyceride deposition [3]. This enables more efficient fatty acid (FA) oxidation, and improved insulin sensitivity in the short term. In the medium term, VLCDs can reduce pancreatic fat and increase insulin secretion, as well as improve the insulin sensitivity of skeletal muscle [4]. The recently published DiRECT trial highlights the effectiveness of these diets [5], showing at 12 months almost half of the participants achieving diabetes remission.

Whilst the combination of improved insulin sensitivity and weight loss should be beneficial to the cardiovascular system [6], this may well not be the case due to the elevation in circulating free fatty acid (FFA) levels that occurs during rapid mobilisation of fat stores. Acute elevation of FFA levels has been shown to impair vascular function in humans [7], and lead to myocardial lipid accumulation and dysfunction in animals [8, 9], with a 3-day extreme VLCD (471 kcal/day) in humans also resulting in impaired diastolic function [10, 11].

The effects of the contemporary VLCDs on myocardial and vascular function in a real-world setting are yet to be established, but are an important consideration given both the historical context of increased cardiovascular risk with VLCDs, and the burgeoning worldwide prevalence of cardiac dysfunction associated with obesity. With these concerns in mind, we sought to investigate in a healthy obese population whether any changes in cardiovascular metabolism and function occur over the course of a modern 8-week VLCD regime, and seek to find whether these dietary interventions have the potential for treatment of obesity-related cardiac dysfunction.

## Methods

Forty volunteers were recruited by poster advertisement, of whom 25 were obese and were allocated to the VLCD, and the remainder were normal weight control subjects. Inclusion criteria were age >18 years, BMI > 30 kg/m^2^ (VLCD group) or <25 kg/m^2^ (control). Exclusion criteria were insulin therapy for diabetes, current or previous history of coronary disease, systolic heart failure, pregnancy, and standard contraindications to magnetic resonance (MR) scanning. The local ethics committee approved the study (REC SC/15/004), and informed written consent was obtained.

### Anthropomorphic and metabolic assessment

Body composition was analysed by bio-impedance (InBody 770, InBody Co. Ltd., South Korea). Non-invasive blood pressure was measured manually (average of 3 supine measurements, Carescape V100, GE Healthcare, Chicago, IL, USA). Fasting venous blood was drawn for circulating biomarkers; glucose, cholesterol, triglycerides, and BNP were analysed by the Oxford University Hospitals NHS Trust laboratory according to standard clinical protocols. FFAs and β-hydroxybutyrate were measured using commercially available colorimetric assay kits (CV <6% and <4%, respectively; Cayman Chemical, MI, USA).

To assess insulin resistance, a mixed meal tolerance test was performed at baseline and 8 weeks (37 g carbohydrate, 12 g protein, and 12 g fat (200 ml Fortisip drink, Nutricia, Schiphol, The Netherlands)), with venous glucose and insulin drawn additionally at 30, 60, and 120 min. Insulin resistance was calculated both using the HOMA-IR model at all 3 time-points ((fasting glucose × fasting insulin)/22.5), and the Matsuda index at baseline and 8 weeks (10,000/√((fasting glucose × fasting insulin) × (mean glucose × mean insulin during meal tolerance test))).

### Magnetic resonance imaging

Left ventricular geometry and systolic function, liver fat, myocardial triglyceride content (MTGC), myocardial energetics (PCr/ATP, 1 and 8 weeks only), regional aortic elastic function, epicardial and abdominal visceral fat were all assessed using a combination of multiparametric MRI and multinuclear spectroscopy (3 Tesla Trio, Siemens, Erlangen, Germany) as described below.

#### Left ventricular geometry and function

LV imaging was retrospectively cardiac-gated and acquired during end-expiratory breath-hold. LV mass, volumes, and ejection fraction were calculated from manual endocardial and epicardial contours (CVI42, Circle Cardiovascular Imaging Inc, Calgary, Canada) [12]. LV mass and EDV are presented indexed to height^1.7^ as is appropriate for this adult population [13]. Strain and strain rates were derived from tissue tracking [14].

#### Myocardial triglyceride content

Myocardial ^1^H-MR spectra were obtained from the inter-ventricular septum [15]. Water-suppressed spectra were acquired to measure MTGC, and spectra without water suppression were used as an internal standard. Spectra were analysed using Matlab and the AMARES algorithm [16].

#### Myocardial energetics

PCr/ATP was derived from a cardiac-gated fully relaxed 1D-CSI acquisition using a 10 cm loop transmit-receive ^31^P surface coil (Pulse Teq, Chobham, UK). The ^31^P coil was matched for each subject using a radio frequency sweeper (Morris Instruments Inc, Ottawa, Canada). In-house script within Matlab was used for analysis.

#### Abdominal visceral fat mass

Abdominal visceral fat area was measured with a 5 mm transverse slice at the level of the 5th lumbar vertebral body, using a water-suppressed turbo spin echo (TSE) sequence, and analysed manually [16].

#### Liver fat content

Hepatic fat was calculated using the Dixon method, and analysed on custom software [17].

#### Epicardial fat volume

Epicardial fat volume was measured by manual contouring of cardiac short axis images, by a blinded investigator (IA) as previously published [18].

#### Aortic distensibility

Regional aortic distensibility (AD) was assessed using an SSFP cine sequence at 2 levels: (1) the ascending thoracic aorta and (2) in the abdomen 12 cm below this slice [7]. During the acquisition of the images, a brachial BP was recorded. AD (mmHg^−1^) was calculated as [(Aortic Area max − Area min)/(Area min)/pulse pressure] × 1000.

### Echocardiography

Diastolic functional parameters were assessed with standard 2D transthoracic echocardiography (Philips Epiq system). These were analysed using Xcelera reporting software (Philips Healthcare). Average *E*/*e*′ values are presented.

### Very low calorie diet

The 25 obese volunteers followed a supervised VLCD (800 kcal per day; 59% carbohydrate, 26% protein, 13% fat, 3% fibre; replacement diet programme, Counterweight Ltd., UK) for 8 weeks before gradual meal reintroduction. Antihypertensive therapy was discontinued at the start of the diet intervention. Volunteers were advised to continue current levels of physical activity, and increase fluid intake with water or calorie-free fluids to ensure adequate hydration.

### Statistics

Statistical analysis was performed using commercial software (SPSS, Chicago, USA). All data is presented as mean ± standard deviation. Homogeneity of distribution was confirmed before determination of statistical significance, either by Chi-squared tests in the case of nominal data, Student’s *t* tests for continuous data (independent for comparison of baseline characteristics), or repeated measures ANOVA (for comparison within individuals at the 3 time points) with the Greenhouse–Geisser correction, with post-hoc tests using the Bonferroni correction. Pearson’s correlation models were used where relevant. Values of *p* < 0.05 were considered as statistically significant.

## Results

**Fig. 1:**
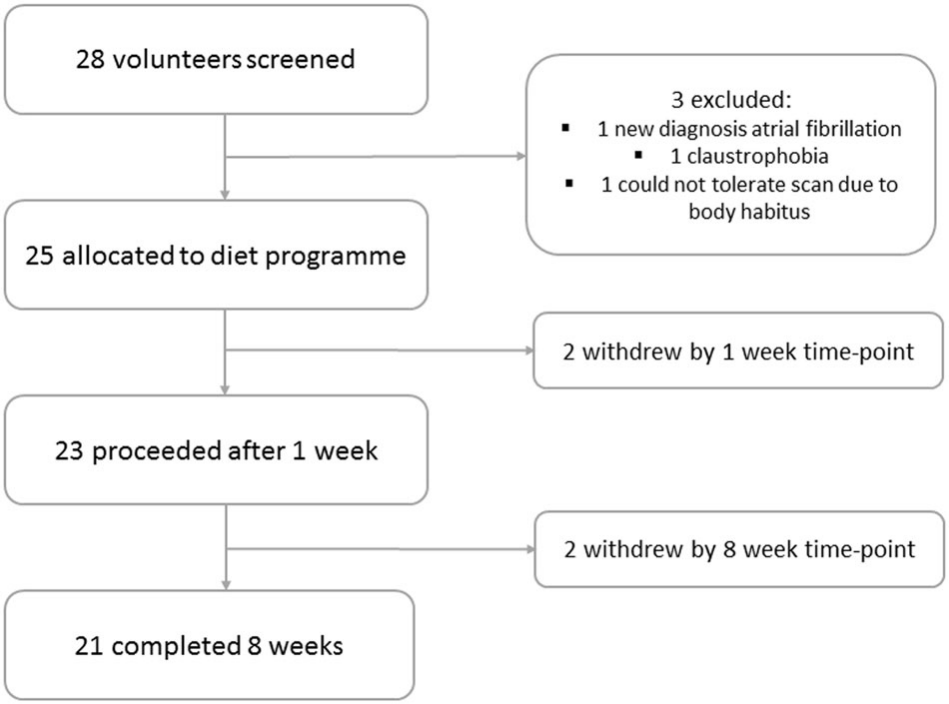
CONSORT diagram demonstrating the study population

Twenty-eight individual volunteers were initially screened for the VLCD study, of whom 1 was excluded due to a new diagnosis of atrial fibrillation, 1 was excluded due to severe claustrophobia, and 1 was unable to tolerate the MRI scans due to body habitus. Of the 25 recruited VLCD volunteers, the majority were female (17/25) (Table 1). Two volunteers withdrew by 1 week, and a further 2 by 8 weeks—there were no significant differences in characteristics in these individuals to those who completed the study (Fig. 1).

### Baseline

#### Anthropometric and metabolic characteristics

VLCD volunteers and controls were well matched for age (study group 49 ± 14 years, controls 45 ± 17 years, *p* = 0.410) and sex (study group 32% male, controls 40% male, *p* = 0.717). As expected, the average BMI of the volunteers was higher than controls (36.8 ± 5.8 vs 24.0 ± 2.4 kg/m^2^, *p* < 0.001). Liver fat was also significantly higher (VLCD 7.4 ± 6.3% vs 0.9 ± 0.6%, *p* < 0.001), and above the threshold for diagnosis of non-alcoholic fatty liver disease (>5%) in 15 individuals. On average, blood pressure in the study group was systolic 135 ± 20 mmHg, and diastolic 89 ± 11 mmHg, with both values significantly higher than controls (*p* < 0.01, Table 1).

**Table 1:** Baseline demographics and anthropomorphic data

	Normal controls *n* = 15	Study population *n* = 25	*p*
**Anthropomorphic**			
BMI (kg/m^2^)	24.0 ± 2.4	36.8 ± 5.8	**<0.001**
Age (years)	45 ± 17	49 ± 14	0.410
Male	6	8	0.717
Systolic blood pressure (mmHg)	121 ± 15	136 ± 19	**0.008**
Diastolic blood pressure (mmHg)	77 ± 11	89 ± 11	**0.004**
**Metabolic parameters**			
Total cholesterol (mmol/l)	4.6 ± 1.0	5.4 ± 1.0	**0.017**
HDL cholesterol (mmol/l)	1.4 ± 0.3	1.6 ± 1.0	0.479
LDL cholesterol (mmol/l)	2.8 ± 0.9	3.1 ± 0.8	0.077
Triglycerides (mmol/l)	1.0 ± 0.5	1.6 ± 0.7	**0.003**
Glucose (mmol/l)	4.8 ± 0.3	5.4 ± 0.9	**0.011**
Insulin (mmol/l)	35 ± 13	94 ± 48	**0.019**
HOMA-IR	1.2 ± 0.5	4.3 ± 5.1	**0.029**
**Medication**			
Antihypertensive therapy	2	4	0.757
ACE inhibitor	1	2	0.834
Angiotensin receptor blocker	0	1	0.418
Calcium channel blocker	2	1	0.308
Statin	1	3	0.545
Metformin	0	2	0.246
**Body fat distribution**			
Body fat mass (kg)	16 ± 7	45 ± 13	**<0.001**
Body fat percentage (%)	23 ± 11	43 ± 8	**<0.001**
Abdominal visceral fat area (cm^2^)	56 ± 45	162 ± 81	**<0.001**
Liver fat (%)	0.9 ± 0.6	7.4 ± 6.3	**0.001**
Epicardial fat (cm^3^)	38 ± 15	102 ± 33	**<0.001**
Myocardial triglyceride content (%)	1.0 ± 0.7	2.2 ± 1.1	**0.003**
**LV geometry**			
LV end-diastolic volume (ml)	146 ± 26	156 ± 27	0.267
LV EDV indexed to height (ml/m^1.7^)	60 ± 9	64 ± 9	**0.211**
LV mass (g)	98 ± 20	114 ± 27	**0.046**
LV mass indexed to height (g/m^1.7^)	40 ± 6	46 ± 9	**0.015**
LV mass-to-volume ratio	0.66 ± 0.06	0.74 ± 0.15	0.056
**Myocardial energetics**			
Myocardial PCr/ATP	2.2 ± 0.2	1.9 ± 0.3	**0.003**
**LV systolic function**			
LV ejection fraction (%)	65 ± 7	66 ± 5	0.408
Peak radial strain (%)	52 ± 10	42 ± 8	**0.002**
Peak circumferential strain (%)	−20 ± 2	−20 ± 2	0.275
Peak longitudinal strain (%)	−16 ± 2	−16 ± 2	0.831
**LV diastolic function**			
LV *E*/*e*′	7.6 ± 1.3	8.6 ± 1.4	**0.022**
Peak radial diastolic strain rate (1/s)	−3.7 ± 1.1	−2.7 ± 1.5	**0.020**
**Aortic distensibility (mmHg)**			
Ascending aorta	4.1 ± 2.7	5.4 ± 3.8	0.284
Distal descending aorta	7.3 ± 2.4	4.9 ± 3.8	**0.026**

Although fasting glucose (5.4 ± 0.9 mmol/l), total cholesterol (5.4 ± 1.0 mmol/l), and plasma triglycerides (1.6 ± 0.7 mmol/l) were within the normal range, they were all higher than controls (all *p* < 0.05, Table 1). The obese VLCD group was significantly more insulin resistant compared to controls (HOMA-IR 4.3 ± 5.1 vs 1.2 ± 0.5, *p* = 0.029).

#### Baseline cardiac function and metabolism

**Fig. 2:**
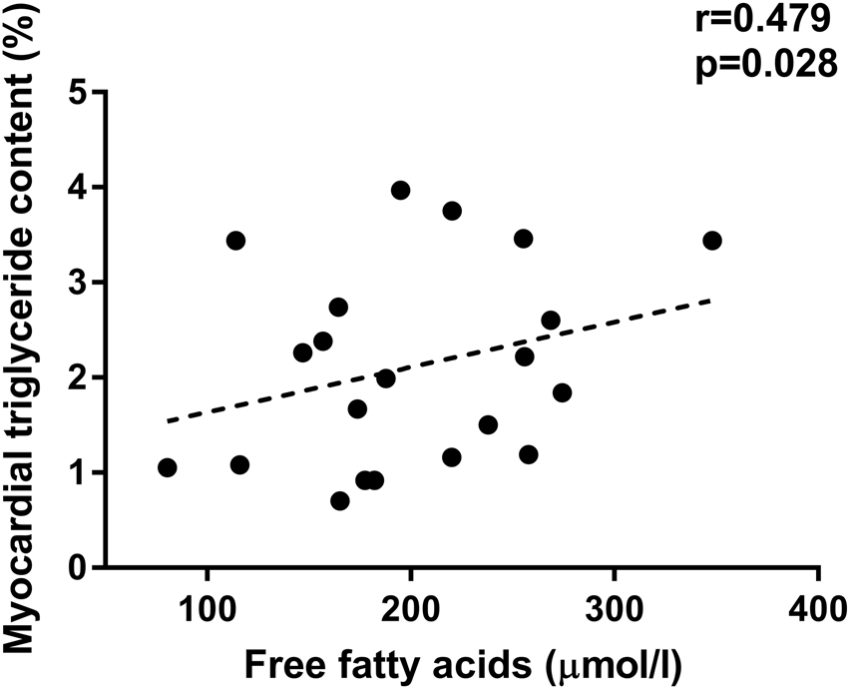
Myocardial triglyceride content correlates with circulating free fatty acids at baseline

The obese VLCD group had higher LV mass (by 15% when indexed to height, *p* = 0.015), two-fold higher myocardial triglyceride levels (*p* = 0.003) and reduced myocardial energetics (PCr/ATP by 14%, *p* = 0.003). There was evidence of subclinical changes in systolic function with reduced radial strain (by 19%, *p* = 0.002). Diastolic function was significantly more impaired in the obese VLCD group at baseline (average *E*/*e*′ 8.4 ± 1.4 vs 7.6 ± 1.3, *p* = 0.022). Importantly, FFA levels correlated with MTGC at baseline (*r* = 0.479, *p* = 0.028, Fig. 2).

#### Aortic elastic function

Although AD was similar to controls in the ascending aorta (*p* = 0.28), abdominal aortic elastic function was significantly impaired (by 33%) in the obese group (4.9 ± 3.8 vs 7.3 ± 2.4 mmHg^−1^, *p* = 0.026).

### The effects of 1 week of VLCD

**Fig. 3:**
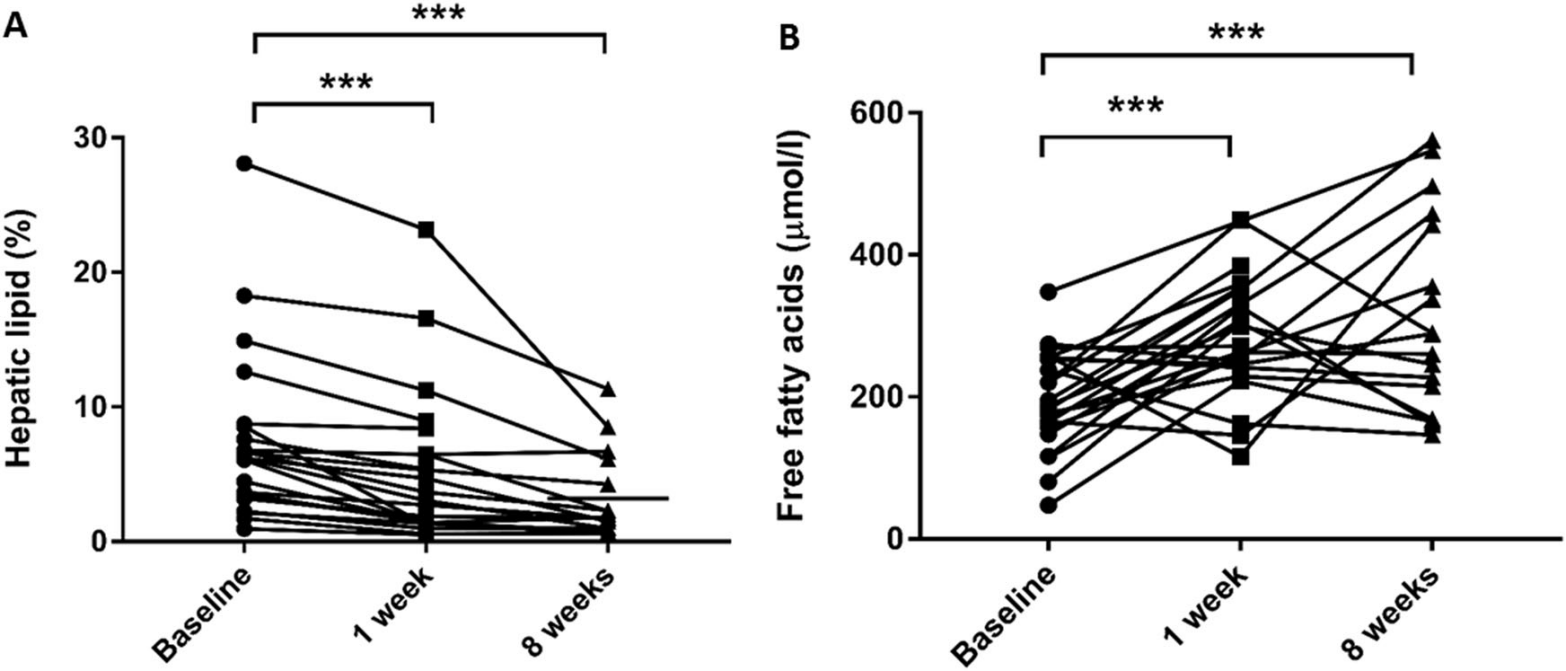
Hepatic lipid (**a**) and free fatty acids (**b**) at baseline, 1 and 8 weeks after initiation of the very low calorie diet (*p < 0.05, ***p < 0.001)

Following 7 days of VLCD, there were small but significant reductions in BMI (1.3 ± 0.5 kg/m^2^), and most fat depots (Table 2). A larger relative fall in hepatic fat occurred (31 ± 24%, *p* < 0.001, Fig. 3a), with significant improvements in fasting glucose (by 13 ± 14%), and insulin sensitivity (HOMA-IR by 42 ± 26%, all *p* < 0.01, Table 2). Despite the withdrawal of antihypertensive medication, systolic blood pressure fell (by 6 ± 9 to 128 ± 14 mmHg, *p* = 0.035).

**Table 2:** Findings at 1 and 8 weeks compared to baseline

	Baseline *n* = 25	1 week *n* = 23	8 weeks *n* = 21
**Anthropometric**			
BMI (kg/m^2^)	36.8 ± 5.8	35.1 ± 5.8**	32.3 ± 5.8**^‡^
Body fat mass (kg)	45 ± 13	42 ± 14**	35 ± 14**^‡^
Body fat percentage (%)	43 ± 8	40 ± 9*	36 ± 10**^‡^
Systolic blood pressure (mmHg)	136 ± 19	128 ± 14*	124 ± 12*
Diastolic blood pressure (mmHg)	89 ± 11	87 ± 16	80 ± 10
**Metabolic parameters**			
Total cholesterol (mmol/l)	5.4 ± 1.0	4.5 ± 1.0**	4.6 ± 1.0**
Triglycerides (mmol/l)	1.6 ± 0.7	1.2 ± 0.6**	1.4 ± 0.9
Beta-hydroxybutyrate (mmol/l)	0.25 ± 0.10	0.55 ± 0.35**	0.46 ± 0.23**
Free fatty acids (µmol/l)	197 ± 71	284 ± 78**	314 ± 143**
Beta natriuretic peptide (mmol/l)	4.8 ± 2.8	3.7 ± 1.7	5.5 ± 3.0*
Glucose (mmol/l)	5.4 ± 0.9	4.7 ± 0.9**	4.8 ± 0.6**
HOMA-IR	4.3 ± 5.1	1.7 ± 1.4*	1.3 ± 0.7*
Matsuda index	19 ± 13	–	24 ± 12**
**Body fat distribution**			
Liver fat (%)	7.4 ± 6.3	5.5 ± 5.0**	3.3 ± 3.0*^†^
Myocardial triglyceride content (%)	2.2 ± 1.1	3.1 ± 1.7*	1.5 ± 0.6*^†^
Epicardial fat (cm^3^)	102 ± 33	91 ± 27*	86 ± 29**^‡^
Abdominal visceral fat area (cm^2^)	162 ± 81	153 ± 93	102 ± 53**^‡^
**LV geometry and function**			
LV end-diastolic volume (ml)	156 ± 27	144 ± 26**	151 ± 27*
LV EDV indexed to height (ml/m^1.7^)	63 ± 9	58 ± 8**	61 ± 8*
LV ejection fraction (%)	66 ± 5	62 ± 6*	64 ± 5
LV mass (g)	114 ± 27	110 ± 23	107 ± 23*
LV mass indexed to height (g/m^1.7^)	46 ± 10	44 ± 8*	43 ± 7
LV strain analysis			
Peak radial strain (%)	42 ± 9	45 ± 11*	48 ± 11
Peak circumferential strain (%)	−20 ± 2	−19 ± 2**	−19 ± 2
Peak longitudinal strain (%)	−16 ± 1	−15 ± 2	−15 ± 3
Peak radial diastolic strain rate (1/s)	−2.7 ± 1.0	−3.2 ± 0.8	−3.2 ± 1.0*
LV average *E*/*e*′	8.6 ± 1.4	9.4 ± 1.7**	7.5 ± 1.5**
Myocardial PCr/ATP	1.9 ± 0.3	–	2.2 ± 0.2*
**Aortic distensibility**			
Ascending aorta	5.4 ± 3.8	4.3 ± 3.4**	5.8 ± 4.6
Distal descending aorta	4.1 ± 2.5	3.9 ± 2.1	5.0 ± 2.8

*p < 0.05 compared to baseline

**p < 0.01 compared to baseline

^†^*p* < 0.05 compared to normal controls (Week 8 data only)

^‡^*p* < 0.01 compared to normal controls (Week 8 data only)

**Fig. 4:**
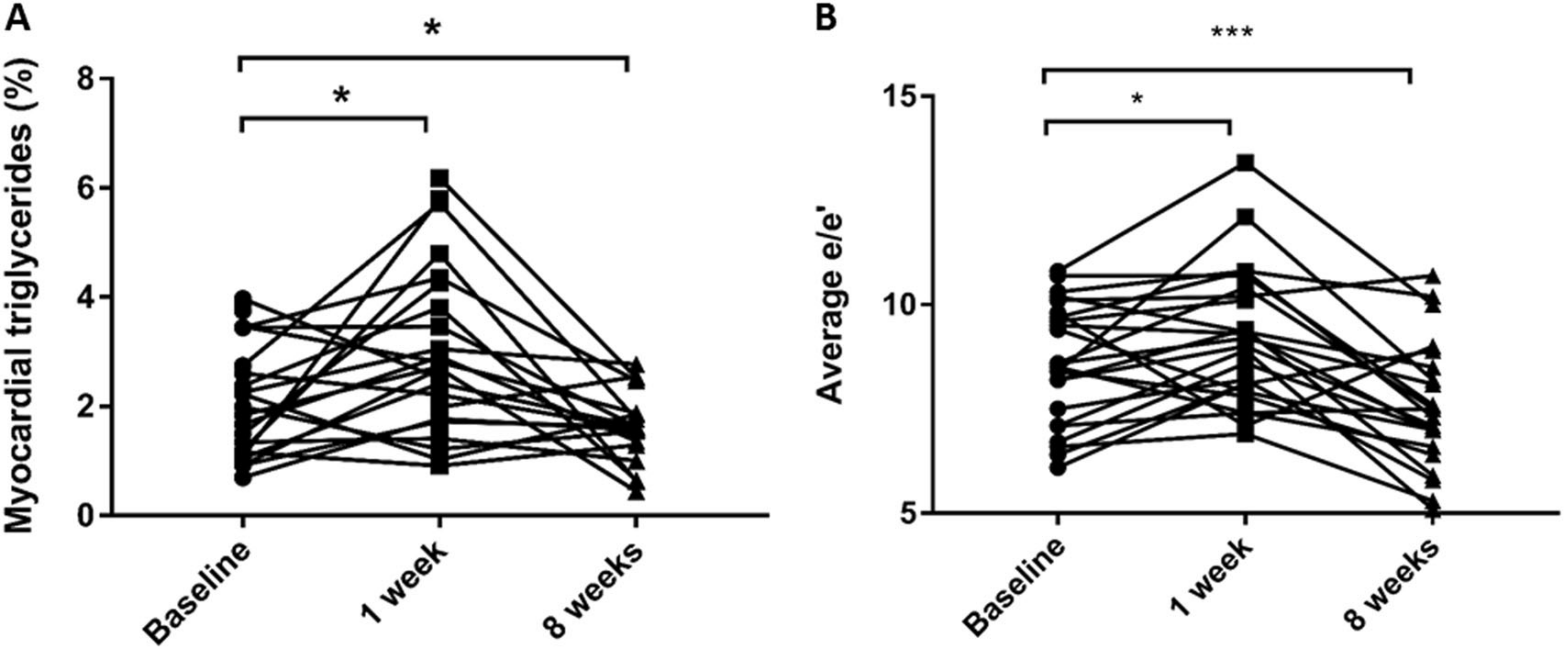
Myocardial triglycerides (**a**) and diastolic function (**b**) at baseline, 1 and 8 weeks after the initiation of the very low calorie diet (*p < 0.05, ***p < 0.001)

Blood β-hydroxybutyrate levels increased (from 0.25 ± 0.10 to 0.55 ± 0.35 mmol/l, *p* = 0.002), supporting adherence to the diet programme, and circulating levels of FAs rose 55% during the first week of VLCD (*p* = 0.017, Fig. 3b). In contrast to other fat depots, MTGC increased by 50% (from 2.1 ± 1.1% to 3.1 ± 1.7%, *p* = 0.030, Fig. 4a). At this time-point, there was a deterioration in both LV systolic function (LVEF 66 ± 5% to 62 ± 6%, *p* = 0.041), and diastolic function (average *E*/*e*′ 8.6 ± 1.4 to 9.4 ± 1.7, *p* = 0.019, Fig. 4b). There was a trend for the change in MTGC to correlate with change in both systolic (LVEF, *r* = −0.43, *p* = 0.08) and diastolic function (*p* = 0.056). Suggesting no change in circulating volume, blood haematocrit did not change from baseline (0.422 ± 0.038 compared to 0.428 ± 0.38, *p* = 0.216). BNP levels remained unchanged during this initial period (4.8 ± 2.8 vs 3.7 ± 1.7 pg/ml, Table 2).

Furthermore, aortic elastic function was reduced from baseline in the ascending aorta (4.3 ± 3.4 from 5.4 ± 3.8 mmHg^−1^, *p* = 0.009).

In summary, after 1 week of VLCD, despite significant metabolic benefits, the already impaired cardiovascular system in obesity deteriorated further, with elevation in MTGC, reduced left ventricular systolic and diastolic function, and further increase in aortic stiffness.

### The effect of 8 weeks of VLCD

After 8 weeks (*n* = 21, average timeframe 53 ± 13 days; all results in Table 2), total body weight continued to fall (by 12% from baseline, *p* < 0.01), with ongoing improvements in insulin sensitivity and lipid profile (Table 2).

Final blood pressure values were lower than baseline (systolic 124 ± 12 mmHg, *p* = 0.035; diastolic 80 ± 10 mmHg, *p* = 0.068), and similar to normal values (control group systolic blood pressure 121 ± 15 mmHg, *p* = 0.192; diastolic 77 ± 11 mmHg, *p* = 0.848).

β-Hydroxybutyrate remained significantly elevated (0.46 ± 0.23 mmol/l compared to 0.25 ± 0.10 mmol/l, *p* = 0.014), indicating likely continued compliance, and FFAs continued to rise (week 8 277 ± 136 vs week 1 168 ± 61 µmol/l, *p* = 0.018, Fig. 3b). However, having initially increased, by 8 weeks MTGC had fallen, and were now significantly lower than baseline (1.5 ± 0.6% compared to 2.2 ± 1.1% at baseline, *p* = 0.050, Fig. 4a). Whilst initially impaired, myocardial energetics improved over the course of the VLCD (PCr/ATP by 14%, *p* = 0.021) and were now similar to normal values (study group 2.2 ± 0.2 compared to controls 2.2 ± 0.2, *p* = 0.54).

By 8 weeks, LV geometry was improved; EDV and mass were significantly lower than baseline, and not statistically different from normal weight controls (EDV 61 ± 8 ml/m^1.7^ compared to control group 60 ± 9 ml/m^1.7^, *p* = 0.403; LV mass 43 ± 7 g/m^1.7^ compared to control group 40 ± 6 g/m^1.7^, *p* = 0.222). Systolic function had returned to normal (LV EF 64 ± 5% compared to 66 ± 5% baseline, *p* = 0.638). Not only had diastolic function improved from the 1 week time-point (*E*/*e*′ 8 weeks 7.5 ± 1.5 vs 1 week 9.4 ± 1.7, *p* < 0.001), but there was a significant improvement from baseline (*E*/*e*′ 8 weeks 7.5 ± 1.5 vs baseline 8.6 ± 1.4, *p* = 0.003, Fig. 4b), with values similar to normal controls (control group 7.6 ± 1.3, *p* = 0.877).

By 8 weeks, AD returned to baseline levels in the ascending aorta (5.8 ± 4.6 mmHg compared to control 4.1 ± 2.7 mmHg, *p* = 0.262), but was still impaired when compared to controls (descending 5.0 ± 2.9 mmHg^−1^ compared to normal 7.3 ± 2.4 mmHg^−1^, *p* = 0.050).

## Discussion

There has recently been a renaissance of VLCDs as a means to reverse the metabolic consequences of obesity. The DiRECT trial, showing effectiveness in diabetes reversal [5], should undoubtedly increase their utilisation further. Despite these metabolic improvements, by elevating FFA levels VLCDs have the potential to cause cardiovascular decline. Here, we demonstrate a transient, early, and significant deterioration in cardiovascular function, with increased MTGC after 1 week of caloric restriction, in contrast to the metabolic improvements seen from the onset of the diet. Despite this early deterioration, by 8 weeks diastolic function had improved to normal values, suggesting that VLCDs may be a therapeutic option for diastolic dysfunction in diabetes and obesity.

### Early cardiovascular deterioration in very low calorie diet

In this study, we highlight that successful weight loss over the course of 2 months in obesity results in substantial cardiovascular benefits. However, we show that initially, there is an increase in myocardial steatosis (by 43%) that is accompanied by reduced LVEF, and diastolic dysfunction (with increased *E*/*e*′). The dominant metabolic processes during profound caloric restriction are the mobilisation of triacylglycerols (TAGs) in adipose tissue and gluconeogenesis by the liver (the main source of glucose during fasting) [19]. TAG is broken down into FAs by three main lipases (adipose triglyceride lipase, hormone-sensitive lipase, and monoglyceride lipase) into FAs which then undergo beta-oxidation. The glycerol backbone of TAG is used as a gluconeogenic substrate and the expression/activation of the main gluconeogenic enzymes (PEPCK-C and G6Pase) during fasting is stimulated by glucagon. As myocardial uptake of FFA is proportional to plasma concentration [20], the relative steatosis is likely to reflect a combination of increased FFA uptake, as well as a degree of impaired intracellular FA oxidation in the context of insulin resistance.

This increase in lipid content is likely to be the underlying cause of functional deterioration, as MTGC has been previously shown to contribute to cardiac dysfunction in human obesity [14]. In addition, the fall in LV end-diastolic volume, in the context of elevated diastolic markers and absence of volume depletion, is likely to represent stiffening of the left ventricle. It is in part reassuring that BNP did not elevate at this time-point; however, this is difficult to interpret given the negative association between obesity and BNP [21] meaning that loss of fat mass itself may lead to an increase in BNP values.

There are a number of mechanisms by which impaired cardiac function can be attributed to increased levels of MTGC. In addition to being a less energy efficient substrate than glucose [22], the oversupply of FFAs beyond the oxidative capacity of the heart leads to the diversion of triglyceride down non-oxidative pathways and accumulation of lipotoxic intermediate metabolites such as ceramide and diacylglycerol. These not only further impair FFA oxidation but also worsen intracellular insulin sensitivity, alter calcium handling [23] and favour accumulation of reactive oxygen species (ROS) [24], all of which impair cardiac function [25].

There is also recent evidence to suggest that myocardial uptake of ketones is both increased in insulin resistance, and is associated with LV dysfunction, suggesting that some of the early functional changes may be related to increased ketones [26].

In a similar fashion to myocardial function, aortic elastic function demonstrates early impairment. As there is evidence both that raised FFAs increase ROS in arterial endothelial cells and impair endothelial function [27], and that artificial elevation of FFAs results in a similar pattern of aortic stiffness [7], it is likely that this is implicated in the vascular stiffening seen.

### Cardiovascular benefits after 8 weeks of VLCD

By 8 weeks, with ongoing and significant reduction in fat mass and improved whole-body insulin sensitivity, myocardial triglyceride levels fall below baseline levels. This suggests that ongoing weight loss enables MTGC to be utilised by the heart, and hence intracellular levels are able to normalise despite ongoing circulating FFA excess.

The normalisation of MTGC is associated with improvements in both systolic and diastolic function, with *E*/*e*′ improving to a level which is both lower than baseline, and similar to normal weight controls. Interestingly, the cohort remains obese in terms of fat mass and BMI after the VLCD intervention, but myocardial energetics and MTGC have returned to normal levels. This, together with the deterioration at 1 week despite weight loss, is consistent with the hypothesis that the energetic and functional impairment seen in obesity may be driven by altered cardiac metabolism. The fall in MTGC despite increased circulating FFAs also suggests that the myocardium may be increasing oxidative capacity for FAs by 8 weeks. As peroxisome proliferator-activated receptor alpha (PPAR-α) is the primary regulator of FA oxidation in the heart [28], and has paradoxically been shown to have a blunted response to FAs in obesity [29], one hypothesis is that increased PPAR-α activity in response to improved insulin resistance and reduced fat mass, is responsible for the decrease in MTGC seen here. It is also interesting to note that despite the echocardiographic evidence of improved diastolic function, BNP levels in fact rise by a small but significant proportion, in keeping with the likely effect of fat mass reduction on BNP metabolism [21].

In summary, after 8 weeks of VLCD, there are continued metabolic improvements accompanied by ongoing reductions in body fat. Despite initial increase in myocardial fat and deterioration in cardiovascular function, by the end of the VLCD, myocardial energy metabolism and diastolic function return to normal. As the cardiac dysfunction is both early and transient, it seems unlikely that it would result in any long-term cardiovascular risk.

## Conclusions

Although VLCDs produce profound and rapid improvements in insulin resistance and hepatic steatosis, they are associated with an early but significant deterioration in cardiac function, metabolism, and aortic elasticity. This impairment is transient, and with a full 8-week programme, improvements in cardiovascular function become evident. Hence, we demonstrate for the first time, that they represent an exciting potential therapeutic strategy for the treatment of obesity-related heart disease.
